# The impact of direct inoculation of ascites into blood culture bottles on ascites culture positivity

**DOI:** 10.1017/ash.2024.84

**Published:** 2024-05-17

**Authors:** Tyler Brehm, Todd Lasco, Mayar Al Mohajer

**Affiliations:** 1 Department of Medicine, Baylor College of Medicine, Houston, TX, USA; 2 CommonSpirit Health, Baylor St. Luke’s Medical Center, Houston, TX, USA; 3 Department of Pathology, Baylor College of Medicine, Houston, TX, USA

## Abstract

**Objective:**

Assess whether direct inoculation of ascites into blood culture bottles would improve ascites culture yield.

**Design:**

Pre-post-study.

**Setting:**

The study was performed at a quaternary academic medical center in Houston, Texas, including all inpatient and emergency department encounters.

**Patients:**

Ascites cultures collected from November 2020 to December 2022 were reviewed and screened for spontaneous bacterial peritonitis. Patients were excluded if a prior ascites culture from the same patient was already included in the study or if there was evidence of secondary bacterial peritonitis.

**Intervention:**

In the pre-intervention period, ascites cultures were collected into a sterile container and inoculated onto/into solid and liquid media. In the post-intervention period, ascites cultures were instead directly inoculated into bioMérieux© blood culture bottles at the bedside.

**Results:**

114 patients met inclusion and exclusion criteria, 61 pre-intervention and 53 post-intervention. Overall ascites culture positivity was 15.8% (18/114), 11.5% (7/61) pre-intervention vs 20.8% (11/53) post-intervention. After adjusting for confounders, the intervention had a trend toward a significant effect on ascites culture positivity (*P* = 0.077). No significant differences were seen in time to positivity, hospital length of stay, or 30-day readmission.

**Conclusions:**

Direct inoculation of ascitic fluid into blood culture bottles led to a small increase in culture yield but lacked statistical significance. This lack of significance may be due to the study being underpowered. Further studies are required to investigate if this is due to procedural inefficiencies (eg, inadequate inoculation volumes) or pragmatic clinical practice considerations (ie, high rates of pre-culture antibiotics).

## Introduction

In patients with decompensated cirrhosis, spontaneous bacterial peritonitis (SBP) is a common and serious complication with an annual incidence of 1.5%–3.5% in outpatients and 10%–30% in hospitalized patients,^1,2^ as well as a 66.2% mortality rate within one year of the first SBP episode.^3^ Antibiotics are often started empirically, but accurate microbiological diagnosis is critical for appropriate treatment given the illness severity of SBP.

Historically, ascites cultures were performed by plating ascitic fluid onto various agars (Blood, MacConkey, and Chocolate) and inoculating into broths (eg, Schaedler, thioglycolates)—with variations by institution and period.^4–10^ However, cultures were negative for these conventional methods more than a third of the time.^4,7^ It was hypothesized that utilizing standard nutrient broth-containing blood culture bottles would improve culture growth rates as they are optimized for low bacterial concentration fluids, as seen in both bacteremia and SBP. Luce et al. demonstrated in patients receiving peritoneal dialysis with peritonitis that direct inoculation into Bactec© blood culture bottles led to higher rates of microbiologic diagnosis when compared to conventional methods (95.9% vs 77.6%; *P* < 0.01).^8^ Runyon et al. found similar results in patients with SBP and culture-negative neutrocytic ascites (CNNA). They demonstrated improved ascites culture yield with direct inoculation of blood culture bottles in a quasi-experimental trial (91% vs 42% for conventional; *P* < 0.01)^9^ and confirmed these findings in a subsequent study with concurrent controls (93% vs 43%; *P* < 0.0001).^4^ Another prospective study by Bobadilla et al. reported similar findings (81% vs 52%; *P* < 0.05).^5^ Siersema et al. further compared direct blood culture inoculation, the conventional method, and a lysis centrifugation method in a prospective cohort, demonstrating superior culture yield with direct inoculation (79% vs 33% vs 46%, respectively; *P* < 0.05).^6^

All studies in the extant literature showed higher culture yield for direct inoculation of blood culture bottles compared to conventional culture methods, with absolute improvement in culture positivity ranging from 22% to 50%.^4–6,9^ However, several limitations may decrease their ecological validity and generalizability. First, all had small sample sizes (*n* = 23–31). Second, most excluded patients with negative cultures and an absolute neutrophil count (ANC) in the 250–500 cells/mL range, which in current medical practice is treated as SBP. Thus, the results do not fully translate to the target population or standard clinical practice. Third, all the studies excluded patients who had received recent antibiotics, which again does not reflect clinical practice where immediate paracenteses are not always performed, and antibiotics are often started empirically prior to ascites culture collection. Last, none of the studies reported demographic information or clinical outcomes of included patients, limiting the generalizability of their findings.

This study aimed to address the above limitations while evaluating if direct inoculation of ascites fluid into bioMérieux© blood culture bottles would increase the percentage of positive ascites cultures in patients with SBP compared to the conventional method.

## Methods

### Study design

This pre- and post-intervention study was completed at Baylor St. Luke’s Medical Center in Houston, Texas, United States—a quaternary academic medical center. The study population included all adult patients with SBP in the inpatient or emergency department settings between November 2020 and December 2022. SBP was defined as an ascitic fluid ANC ≥ 250 cells/mL in patients with cirrhosis and no identifiable causes of secondary peritonitis. It was approved by the Institutional Review Board of Baylor College of Medicine (IRB H-51376).

### Intervention

This study examined the effect of changing the method of ascites culture collection on ascites culture positivity. In the pre-intervention period, ascitic fluid was collected in a sterile container and centrifuged at 1500 revolutions per minute for 20 minutes. The sediment was resuspended in 1.5 mL of supernatant and plated onto Blood, Chocolate, MacConkey agars, and Schaedler broth. Plates were incubated for 48 hours at 34–36°C and 5% CO_2_ (Blood and Chocolate) or 48 hours at 34–36°C in an atmospheric incubator (MacConkey and Schaedler)—initial read for culture growth occurred 18–24 hours post-plating. Cultures were finalized if no growth was observed by 48 hours.

In the post-intervention period, ascitic fluid was directly inoculated into one each of bioMérieux© aerobic and anaerobic blood culture bottles at the bedside and, upon laboratory receipt, immediately incubated inside the blood culture BacT/Alert VIRTUO detection system (BioMérieux©) for 48 hours. The bottle(s) were removed upon microbial growth, and blood culture broth was plated onto Blood, Chocolate, and MacConkey agars. The medical staff was informed of the change in ascites culture collection methodology via printed and electronic memos and face-to-face communication, along with a notification that the microbiology laboratory would reject improperly collected samples.

### Patient identification and chart review

All ascites cultures in the study period were identified via the Reporting Workbench (RWB) in Epic (Epic Systems Corporation, Verona, Wisconsin, United States). Ascites cultures were selected if classified as “Body Fluid Culture” with a reported source of “ascites,” “ascitic,” “paracentesis,” or “abdomen.” Cultures were included if the ascites fluid was collected between November 1, 2020 to October 31, 2021 (pre-intervention), or December 1, 2021 to December 31, 2022 (post-intervention) and had an ANC ≥ 250 cells/mL. November 2021 was excluded as a washout period as it was the intervention implementation month. Patients were additionally excluded if they had a prior included ascites culture during the study period, ascites from an etiology other than cirrhosis, peritoneal dialysis or indwelling peritoneal catheter, abscess or fluid collection on imaging, or a surgical finding of a perforated viscus.

The primary outcome was the percentage of positive ascites cultures. Secondary outcomes were days of antimicrobial therapy (DOT), time to ascites culture positivity, days of hospitalization, 30-day readmission rate, and 30-day mortality rate.

DOT was defined as the aggregate sum of days of antimicrobials given to an individual patient as documented within the electronic health record (EHR), within either 72 hours prior to ascites culture collection or up to 7 days after and excluding antibiotics administered for prophylaxis. Time to ascites culture positivity was defined as the time from ascites culture collection to the time of first antimicrobial growth.

Patient demographics and clinical characteristics were collected from the EHR. Demographics collected were age, gender, race, and ethnicity. Clinical features included cirrhosis etiology, the MELD (model for end-stage liver disease, Organ Procurement and Transplant Network) score, and SBP risk factors including SBP prophylaxis before ascites culture, history of SBP, history of variceal hemorrhage, ascitic fluid total protein <1 g/dL, and the use of proton pump inhibitors within 30 days.

Confounding variable data collected included pre-culture antibiotic administration, location of paracentesis, and culture bottle inoculation volumes. Pre-culture antibiotics and the location of paracentesis were collected from the EHR. The locations of paracentesis were the emergency department, interventional radiology (IR) suite, and the intensive-care unit. Data for inoculation volumes were collected from all ascites cultures obtained post-intervention from December 2021 to May 2022, including patients with and without SBP, via the bioMérieux© instrument data management platform (MYLA). Based on manufacturer recommendations, inoculation volumes were categorized as underfilled (<8 mL), appropriately filled (8–10 mL), or overfilled (>10 mL).^4–6,9^ Any missing volumes were excluded from the analysis.

Organisms from positive ascites cultures were recorded and EHR analyzed by the primary author (TB) to determine if antibiotic regimens for SBP were escalated, de-escalated, or unchanged by culture results. Charts were reviewed for antibiotic changes which occurred after culture results but within 7 days of culture collection and which were documented to be addressing SBP. The final antibiotic regimen was recorded for purposes of determining escalation or de-escalation, in cases where multiple changes occurred. Escalation referred to the addition of more antibiotics (eg, adding vancomycin to an initial regimen of ceftriaxone only) or broadening spectrum of coverage (eg, changing ceftriaxone to cefepime or ertapenem). De-escalation referred to the removal of antibiotics or narrowing spectrum of coverage.

### Statistical analysis

Power analysis was performed prior to data collection; incidence rates for ascites culture positivity were estimated to be 20% for pre-intervention versus 50% for post-intervention collection methods, respectively. The difference of 30% was a conservative estimate based on prior reported data.^4–6,9^ Given an alpha level of 0.05 and a power of 80%, 44 patients in the pre-intervention group and 35 in the post-intervention group were required.

The Welch’s *t* test (or Wilcoxon rank sum test as appropriate) was used for continuous variables, while χ^2^ (or Fisher’s exact as appropriate) was used for categorical variables. Five multiple regression models (Model 1-Model 5) were employed to assess factors associated with the study outcomes (culture positivity, time to positivity, days of hospitalization, 30-day readmission rate, and mortality). Independent factors included the study intervention, patient demographics, clinical characteristics, and paracentesis location. Patients with missing data were excluded from the regression models. Linear regression was used for continuous outcomes (Model 2, Model 3), while logistic regression was used for dichotomous outcomes (Model 1, Model 4, and Model 5). Fitness was assessed via R^2^/R^2^ adjusted for linear regressions (Nagelkerke R^2^ if negative) and Tjur R^2^ for logistic regressions. Significance was defined as a *P* value <0.05. Statistical analysis was performed using R version 4.2.1 (R Foundation for Statistical Computing, Vienna, Austria).

## Results

### Patient screening

**Figure 1.**
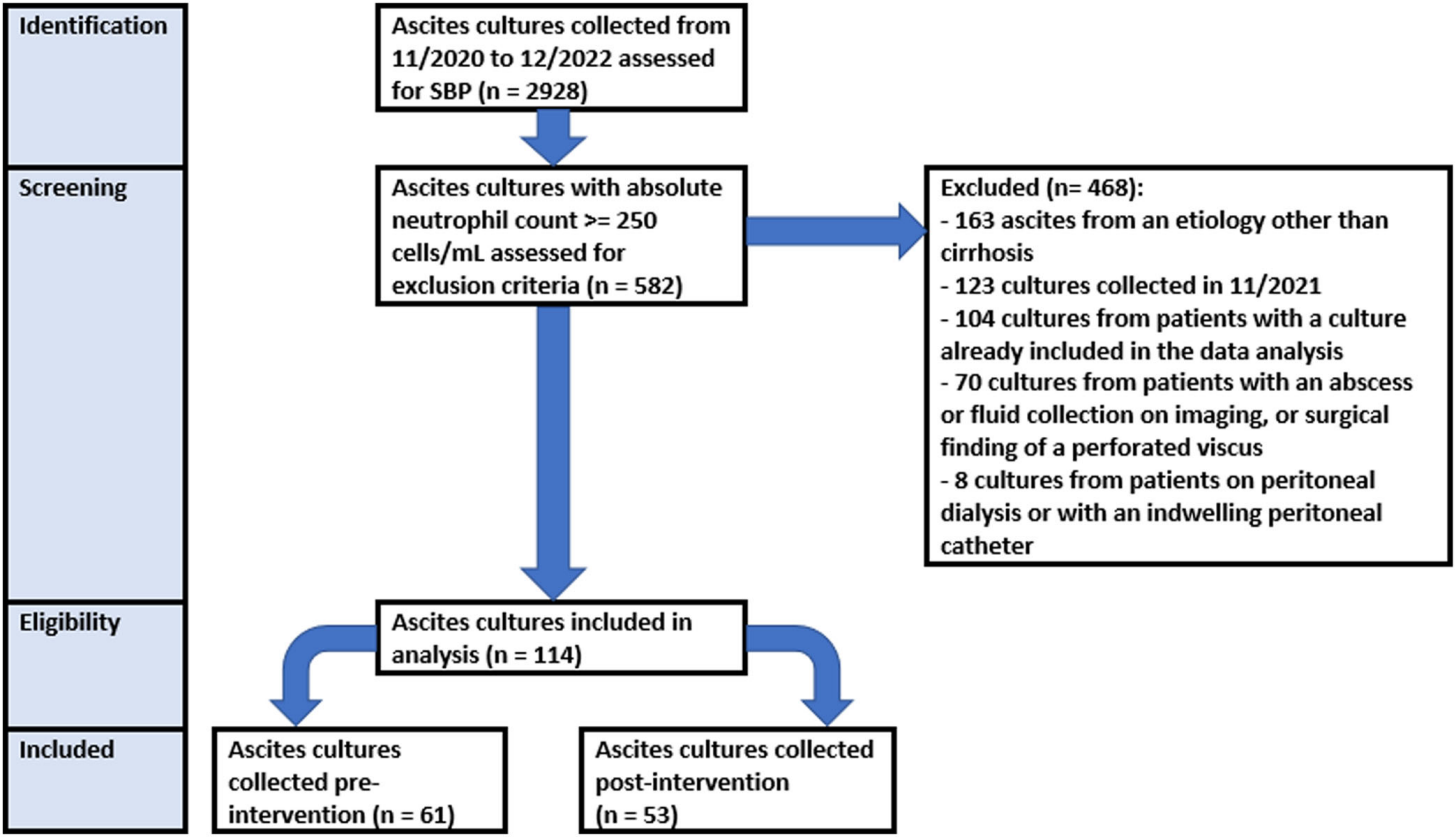
Study flow chart. SBP = spontaneous bacterial peritonitis.

A total of 2,928 ascites culture samples were identified from November 2020 to December 2022. Of these, 582 had an ANC ≥ 250 cells/mL. 468 of these 582 were then excluded (Figure 1). 114 patients with SBP remained and were included in the subsequent analysis (61 pre-intervention and 53 post-intervention).

### Patient characteristics

Patient characteristics are presented in Table 1. There were no statistical differences between pre- and post-intervention groups in terms of age, gender, ethnicity, or race. There was a statistically higher proportion of patients with ethanol-induced cirrhosis in the pre-intervention group compared to the post-intervention group (65.6% vs 41.5%, *P* = 0.010). There were no statistical differences between pre- and post-intervention groups regarding SBP risk factors, SBP prophylaxis, or MELD scores.

**Table 1.** Characteristics of patients with spontaneous bacterial peritonitis

Characteristic	Overall (*n* = 114)	Pre-intervention (*n* = 61)	Post-intervention (*n* = 53)	*P* value
Median Age (Q1-Q3)–Years	60 (49–66)	60 (48–68)	59 (49–64)	0.714
Gender–Number (%)				0.189
Female	57 (50.0)	27 (44.3)	30 (56.6)	
Male	57 (50.0)	34 (55.7)	23 (43.4)	
Ethnicity–Number (%)				0.654
Hispanic	39 (34.2)	22 (36.1)	17 (32.1)	
Non-Hispanic	75 (65.8)	39 (63.9)	36 (67.9)	
Race–Number (%)^a^				0.818
White or Caucasian	91 (85.0)	50 (86.2)	41 (83.7)	
Black or African American	13 (12.1)	7 (12.1)	6 (12.2)	
Asian	1 (0.9)	1 (1.7)	0 (0.0)	
Native Hawaiian or Other Pacific Islander	1 (0.9)	0 (0.0)	1 (2.0)	
American Indian or Alaska Native	1 (0.9)	0 (0.0)	1 (2.0)	
Cirrhosis Etiology–Number (%)^b^				0.010
Ethanol	62 (54.4)	40 (65.6)	22 (41.5)	
Non-Alcoholic Steatohepatitis	35 (30.7)	15 (24.6)	20 (37.7)	
Hepatitis C	17 (14.9)	10 (16.4)	7 (13.2)	
Autoimmune Hepatitis	7 (6.1)	4 (6.6)	3 (5.7)	
Cryptogenic	5 (4.4)	1 (1.6)	4 (7.5)	
Primary Biliary Cholangitis	5 (4.4)	3 (4.9)	2 (3.8)	
Hepatitis B	2 (1.8)	1 (1.6)	1 (1.9)	
Primary Sclerosing Cholangitis	1 (0.9)	1 (1.6)	0 (0.0)	
Methotrexate-induced	1 (0.9)	1 (1.6)	0 (0.0)	
SBP Prophylaxis Before Ascites Culture–Number (%)	16 (14.0)	12 (19.7)	4 (7.6)	0.083
Prior episode of SBP–Number (%)	26 (22.8)	18 (29.5)	8 (15.1)	0.067
Prior History of Variceal Hemorrhage–Number (%)	21 (18.4)	9 (14.8)	12 (22.6)	0.336
Ascitic Fluid total protein < 1 g/dL in Prior Ascitic fluid sample–Number (%)	25 (21.9)	13 (21.3)	12 (22.6)	1.000
Use of PPIs within 30 Days–Number (%)	64 (56.1)	33 (54.1)	31 (58.5)	0.637
Median MELD (Q1-Q3)	26 (22–33)	26 (22–32)	26 (21–33)	0.950
Paracentesis Location–Number (%)				0.584
Interventional Radiology Suite	82 (71.9)	42 (68.9)	40 (75.5)	
Emergency Department	10 (8.8)	5 (8.2)	5 (9.4)	
Intensive-Care Unit	22 (19.3)	14 (23.0)	8 (15.1)	

Q1-Q3 = Quartile 1 to Quartile 3; SBP = spontaneous bacterial peritonitis; PPI = proton pump inhibitor; MELD = model for end-stage liver disease (Organ Procurement and Transplant Network model).

a For statistical comparison purposes, races were combined into White or Caucasian versus Non-White or Caucasian due to low sample size.

b For purposes of statistical comparison, cirrhosis etiologies were combined into cirrhosis secondary to ethanol versus cirrhosis not secondary to ethanol (due to low sample size). If a patient had a cirrhosis etiology attributed to more than one etiology (eg, hepatitis C and ethanol), they were counted as one patient in each category.

Therapeutic antibiotics (not prescribed for SBP prophylaxis) were administered before ascites culture collection in 62.3% vs 60.4% of the pre- and post-intervention group, respectively (*P* = 0.834). There was no difference in the proportions of paracentesis locations between groups (Table 1, *P* = 0.584).

### Primary outcome

Ascites culture positivity was 15.8% (18/114) for the entire cohort and was similar between pre- and post-intervention groups (Table 2, 11.5% [7/61] vs 20.8% [11/53], *P* = 0.205). After adjusting for confounders, there was a trend toward significance in ascites culture positivity after the intervention (Supplemental Table 1, Model 1, *P* = 0.077). Variables significantly associated with ascites culture positivity included the location of paracentesis (non-IR locations vs IR, *P* = 0.029), prior variceal hemorrhage (*P* = 0.044), and ascitic fluid protein ≤ 1 g/dL (*P* = 0.004).

**Table 2.** Primary and secondary outcomes of patients with spontaneous bacterial peritonitis (Univariate analysis)

Outcome	Pre-intervention (*n* = 61)	Post-intervention (*n* = 53)	*P* value
Culture Positivity–Number (%)	7 (11.5)	11 (20.8)	0.205
Median days of antibiotic therapy–Days (Q1-Q3)	9.0 (6.0–22.0)	11.0 (8.0–18.0)	0.789
Median Days of Hospitalization–Days (Q1-Q3)	14.0 (9.0–23.0)	13.0 (8.0–29.0)	0.932
Median time to positivity–Hours (Q1-Q3)^a^	25.9 (23.8–37.2)	41.5 (38.4–45.8)	0.057
Readmission Within 30 days–Number (%)	18.0 (29.5)	16 .0 (30.2)	1.000
30-Day Mortality–Number (%)	10.0 (16.4)	6 (11.3)	0.590

Q1-Q3 = Quartile 1 to Quartile 3.

a *n* = 7 and 11 for pre- and post-intervention groups, respectively, for this secondary outcome as it only compares patients with positive cultures.

There were 48 positive cultures out of the 1704 samples collected post-intervention from December 2021 to May 2022. These positive samples were classified as underfilled (54.2%), appropriately filled (14.6%), and overfilled (31.3%). The median inoculation volume was 7 mL for positive cultures (IQR 5–12 mL) versus 11.0 mL for negative cultures (IQR 10–13 mL, *P* < 0.001).

### Secondary outcomes

The time to positivity was longer in the post-intervention period (25.9 hours pre-intervention vs 41.5 hours post-intervention, *P* = 0.057, Table 2) but did not achieve statistical significance. Similarly, no statistical difference was observed in the length of stay or 30-day readmission between the pre- and post-intervention groups (Table 2). Results were similar after adjusting for confounders (Supplemental Table 1, Models 2, 3, and 4). We could not report the regression results for mortality (Model 5) as the model did not converge due to the small number of patients who met this outcome.

### Microbiological results

Organisms for the 18 positive cultures are reported in Table 3. Notably, 33.3% (6/18) of positive cultures grew organisms for which ceftriaxone (commonly recommended empiric therapy for SBP) is not recommended. There was no statistical difference between those with positive versus negative culture results in proportion of antibiotic regimen adjustment (*p* = 0.176, Table 4). Regimen changes are detailed in Supplemental Table 2.

**Table 3.** Organism and antibiotic regimen adjustment in patients with spontaneous bacterial peritonitis and positive ascites cultures

Patient #	Organism(s)	Antibiotic regimen adjustment
Pre-intervention		
Patient 1	*Klebsiella pneumoniae*	No change
Patient 2	*Klebsiella pneumoniae*	De-escalated
Patient 3	*Enterobacter cloacae*	No change
Patient 4	*Klebsiella pneumoniae*	No change
Patient 5	*Escherichia coli; Streptococcus anginosus*	Escalated
Patient 6	*Morganella morganii*	De-escalated
Patient 7	*Candida tropicalis*	Escalated
Post-intervention		
Patient 8	*Vancomycin-resistant Enterococcus faecium*	Escalated
Patient 9	*ESBL Escherichia coli*	Escalated
Patient 10	*Serratia marcescens*	No change
Patient 11	*Pseudomonas aeruginosa*	Escalated
Patient 12	*Streptococcus parasanguinis*	No change
Patient 13	*Escherichia coli*	De-escalated
Patient 14	*ESBL Escherichia coli*	De-escalated
Patient 15	*Escherichia coli*	De-escalated
Patient 16	*Klebsiella pneumoniae*	De-escalated
Patient 17	*Beta-hemolytic streptococcus group C*	De-escalated
Patient 18	*Klebsiella pneumoniae*	De-escalated

ESBL = extended spectrum beta-lactamase producing. Escalation refers to the addition of more antibiotics or change to a broader spectrum of coverage, while de-escalation refers to the removal of antibiotics or change to a narrower spectrum of coverage.

**Table 4.** Antibiotic regimen adjustment in spontaneous bacterial peritonitis patients with positive versus negative cultures

Antibiotic regimen adjustment	Overall (*n* = 114)	Positives cultures (*n* = 18)	Negative cultures (*n* = 96)
No change–Number (%)	53 (46.5)	5 (27.8)	48 (50.0)
Escalation–Number (%)	28 (24.6)	5 (27.8)	23 (24.0)
De-escalation–Number (%)	33 (28.9)	8 (44.4)	25 (26.0)

Cultures refer to ascites cultures. Escalation refers to the addition of more antibiotics or change to a broader spectrum of coverage, while de-escalation refers to the removal of antibiotics or change to a narrower spectrum of coverage.

## Discussion

This study demonstrated an absolute increase in culture positivity (9.3%) with a trend toward statistical significance after direct inoculation of ascites fluid into blood culture bottles. There are several plausible explanations for why statistical significance was not observed compared to other studies.^4–6,9^ First, this study may have been underpowered. Although the sample size was higher than the projected amount by power analysis, percentage of positive cultures were significantly lower for both methods than the reported literature.^4–6,9^ Thus, the true difference in culture positivity in this population may be lower than the estimated 30%.

The lower culture positivity percentage, in turn, may be due to the higher rates of pre-culture antibiotic treatments (61.4% vs 0% in the three studies that commented on pre-antibiotic use).^4,6,9^ It also may be due to a difference in the underlying prevalence of SBP—this study set a minimum ANC of 250 for both SBP and CNNA, less specific than the minimum ANC of 500 set in prior studies for CNNA.^4–6,9^ This ANC cutoff was chosen in line with American Association for the Study of Liver Diseases (AASLD) guidelines^11^ but does increase the likelihood of inclusion of patients without microbiological evidence of SBP. It could also be due to finalizing cultures at 48 hours and missing slower growing organisms.

An additional reason for the lack of significant improvement with bedside inoculation of blood culture bottles could be improper sample collection. When using bioMérieux© blood culture bottles, the manufacturer reports that the optimal inoculation volume is 8–10 mL, and the culture yield decreases when filled past 10 mL. In a larger sample set looking at all ascites cultures collected in a 6-month period (not exclusively patients with SBP), only 22.1% of culture bottles had volumes in the optimal range. When comparing positive and negative cultures, there was, on average, significantly lower inoculation volume in positive cultures. This suggests a role of human factors that should be addressed to optimize positivity rates.

For the secondary outcomes, no significant differences were observed. This could be related to the absence of a statistically significant change in the primary outcome or the study’s lack of power to detect differences in secondary outcomes. Of note, there was a trend toward increased time to positivity with an average increase of ∼16 hours, although this was not significant on the initial analysis *(P* = 0.057) or after adjusting for the other independent variables *(P* = 0.182). This difference may be because bioMérieux© recommends the addition of defibrinated horse blood for non-blood culture samples, which was not part of our institution’s lab protocol at the time of this study. Notably, this additive assists in the growth of fastidious organisms (eg, *Haemophilus influenza*) and it is unclear whether it impacts bacterial growth times and yield of non-fastidious organisms. Additionally, there may be a delay in the inoculation of ascitic fluid into the blood culture bottles at the bedside, depending on procedural logistics. Unfortunately, this was not a reported measurement that could be analyzed at our institution.

A proposed benefit of increased culture yield is the appropriate tailoring of antibiotic regimens, both in terms of de-escalation (for antibiotic stewardship) and escalation (to prevent progression of the infection). We did not demonstrate a statistically significant change in antibiotic regimen de-escalation. However, we were not powered for this outcome and the 18.4% absolute increase in de-escalation (53.6% relative increase) in culture-positive patients indicates there may be some validity to this concept. Cultures also captured a significant portion of organisms resistant to standard empiric therapy (33.3%), all of which were adjusted to an appropriate regimen after culture results. Our study has several limitations. First, it has a quasi-experimental approach without a control group, making it difficult to assess the role of external factors that could have confounded the results. We have attempted to adjust for that by including patients’ demographics and characteristics in multiple regression models. Second, examination of confounding variables revealed a large proportion of ascites cultures were inoculated with inappropriate volumes (77.9%), which can be reasonably presumed to lower the percentage of positive cultures. Last, as a single-center study, it may not accurately reflect patient populations of other practice settings.

This study has several advantages. First, the sample size is substantially larger than most studies reported in the literature (*n* = 114 vs *n* = 23–31).^4–6,9^ Second, the study population more accurately reflects patients observed in clinical practice. Specifically, it includes patients with CNNA and an ANC of 250–500 cells/uL—an SBP variant treated the same as culture-positive SBP but excluded from prior studies. Our study also has a high proportion of patients treated with antibiotics pre-culture collection (61.4% vs 0% in other studies),^4,6,9^ which, due to the morbidity associated with delay of antibiotics and logistic limitations for obtaining an expedient paracentesis, is likely a factor that applies to other healthcare systems as well. We also adjusted for demographics and clinical characteristics. Although there were no noteworthy differences between pre- and post-intervention groups, these data are absent from prior related studies.^4–6,9^ Thus, this study provides novel data that may be used as a baseline for comparison in future investigations. For outcomes in particular, prior studies imply that improved microbiologic diagnosis will improve patient-centered outcomes, but this has yet to be explicitly demonstrated. This study reports no improvements in clinically relevant outcomes.

Our study is the first to demonstrate no statistically significant improvement in ascites culture positivity with a change to direct inoculation of blood culture bottles at the bedside. This finding may be due to procedural errors (eg, inappropriate inoculation volumes) or the study being underpowered (for a population with ∼60% pre-culture antibiotics), but both possibilities highlight important considerations for any institution planning to transition methods of ascites culture collection. Our results suggest that if direct inoculation improves culture yields, the effect size is significantly smaller than previously reported when applied to realistic clinical settings. Future investigations are needed to optimize ascitic culture yield and determine if this improves patient- and hospital-centered outcomes.

## Supporting information

Brehm et al. supplementary material 1 Brehm et al. supplementary material

Brehm et al. supplementary material 2 Brehm et al. supplementary material

## Data Availability

Based on our IRB protocol, data collected and utilized in this study will not be publicly available.

## Supplementary material

The supplementary material for this article can be found at https://doi.org/10.1017/ash.2024.84
