# Supplementary material for: The impact of direct inoculation of ascites into blood culture bottles on ascites culture positivity
Source: Antimicrob Steward Healthc Epidemiol. 2024 May 17;4(1):e85. doi: 10.1017/ash.2024.84 (PMC11106731; doi:10.1017/ash.2024.84)
Supplement: Brehm et al. supplementary material 1 — Brehm et al. supplementary material [file S2732494X24000846sup001.docx]

**Supplemental Table 1. Multiple Regression Models for Study Outcomes**

|  | **Model 1: Culture Positivity** | | **Model 2: Time**  **to Positivity** | | **Model 3: Days of Hospitalization** | | **Model 4: 30-Day Readmission Rates** | |
| --- | --- | --- | --- | --- | --- | --- | --- | --- |
| *Predictors* | *Odds Ratio* | *P value* | *Estimate* | *P value* | *Estimate* | *P value* | *Odds Ratio* | *P value* |
| **(Intercept)** | 0.00 | 0.044 | 51.44 | 0.083 | 12.36 | 0.539 | 7.45 | 0.161 |
| **Intervention** | 3.94 | 0.077 | 10.23 | 0.182 | 7.41 | 0.252 | 1.22 | 0.683 |
| **Age** | 1.04 | 0.206 | -0.25 | 0.353 | -0.16 | 0.558 | 0.98 | 0.252 |
| **Gender (Male vs. Female)** | 1.45 | 0.607 | 10.84 | 0.178 | 3.04 | 0.643 | 0.88 | 0.791 |
| **Ethnicity (Hispanic/Latino vs. Not Hispanic/Latino)** | 0.49 | 0.299 | -2.91 | 0.825 | 1.03 | 0.881 | 0.50 | 0.166 |
| **Race (Caucasian vs. Non-Caucasian)** | 0.55 | 0.563 | 5.21 | 0.539 | 3.93 | 0.672 | 0.59 | 0.424 |
| **Paracentesis Location (Other vs. IR)** | 5.22 | 0.029 | -5.79 | 0.367 | 5.49 | 0.449 | 1.04 | 0.936 |
| **Cirrhosis Etiology (Ethanol vs. Non-Ethanol)** | 1.90 | 0.453 | -12.01 | 0.321 | 5.62 | 0.446 | 0.88 | 0.813 |
| **SBP Prophylaxis Before Ascites Culture** | 0.75 | 0.774 | -15.49 | 0.345 | 2.05 | 0.854 | 3.27 | 0.163 |
| **Prior Episode of SBP** | 3.82 | 0.112 | 0.43 | 0.968 | -2.16 | 0.816 | 0.47 | 0.311 |
| **Prior History of Variceal Hemorrhage** | 4.78 | 0.044 | 3.73 | 0.499 | 14.81 | 0.069 | 0.95 | 0.935 |
| **Ascitic Fluid Total Protein < 1 g/dL** | 7.11 | 0.004 | -7.80 | 0.490 | -1.34 | 0.859 | 0.34 | 0.091 |
| **Use of PPIs Within 30 Days** | 0.39 | 0.157 | 0.18 | 0.979 | 7.91 | 0.215 | 0.58 | 0.235 |
| **Observations** | 107 | | 17 | | 107 | | 105 | |
| **R^2^** | 0.265 | | 0.856 / 0.426 | | 0.101 / -0.014 | | 0.098 | |

*IR: Interventional Radiology; SBP: spontaneous bacterial peritonitis; PPI: proton pump inhibitor; R^2^: coefficient of determination.*

*R^2^ values are reported as Tjur R^2^ for logistic regression (Models 1 and 4) and R^2^/R^2^ adjusted for linear regression (Models 2 and 3).*

*Model 5 (Mortality) results were not reported as the model did not converge.*
