# Supplementary material for: The impact of direct inoculation of ascites into blood culture bottles on ascites culture positivity
Source: Antimicrob Steward Healthc Epidemiol. 2024 May 17;4(1):e85. doi: 10.1017/ash.2024.84 (PMC11106731; doi:10.1017/ash.2024.84)
Supplement: Brehm et al. supplementary material 2 — Brehm et al. supplementary material [file S2732494X24000846sup002.docx]

**Supplemental Table 2. Organism and Antibiotic Regimens in Patients with Spontaneous Bacterial Peritonitis and Positive Ascites Cultures**

| **Patient #** | **Organism(s)** | **Pre-Culture Results**  **Antibiotic Regimen** | **Post-Culture Results**  **Antibiotic Regimen** |
| --- | --- | --- | --- |
| **Pre-Intervention** |  |  |  |
| Patient 1 | *Klebsiella pneumoniae* | Ceftriaxone | Ceftriaxone |
| Patient 2 | *Klebsiella pneumoniae* | Piperacillin-tazobactam, Vancomycin, Micafungin | Piperacillin-tazobactam |
| Patient 3 | *Enterobacter cloacae* | Ceftriaxone | Ceftriaxone |
| Patient 4 | *Klebsiella pneumoniae* | Ceftriaxone | Ceftriaxone |
| Patient 5 | *Escherichia coli; Streptococcus anginosus* | Ceftriaxone | Piperacillin-tazobactam → meropenem |
| Patient 6 | *Morganella morganii* | Cefepime, Vancomycin | Piperacillin-tazobactam |
| Patient 7 | *Candida tropicalis* | None | Fluconazole |
|  |  |  |  |
| **Post-Intervention** |  |  |  |
| Patient 8 | *Vancomycin-resistant Enterococcus faecium* | Vancomycin, Meropenem, Micafungin | Linezolid, Meropenem, Micafungin |
| Patient 9 | *ESBL Escherichia coli* | Cefepime, Vancomycin | Meropenem →  Ertapenem |
| Patient 10 | *Serratia marcescens* | Ceftriaxone | Ceftriaxone |
| Patient 11 | *Pseudomonas aeruginosa* | Meropenem | Ceftazidime |
| Patient 12 | *Streptococcus parasanguinis* | Ceftriaxone | Ceftriaxone |
| Patient 13 | *Escherichia coli* | Cefepime, Metronidazole, Vancomycin | Ceftriaxone |
| Patient 14 | *ESBL Escherichia coli* | Ceftriaxone →  Meropenem | Meropenem →  Ertapenem |
| Patient 15 | *Escherichia coli* | Meropenem, Micafungin, Vancomycin | Piperacillin-tazobactam |
| Patient 16 | *Klebsiella pneumoniae* | Ceftriaxone →  Piperacillin-tazobactam → Meropenem | Ciprofloxacin |
| Patient 17 | *Beta-hemolytic streptococcus group C* | Piperacillin-tazobactam, Metronidazole, Vancomycin | Ceftriaxone→  Amoxicillin-clavulanate |
| Patient 18 | *Klebsiella pneumoniae* | Piperacillin-tazobactam | Ceftriaxone |

*Antibiotics separated by commas were given concurrently. Antibiotics separated by arrows (→) were given at sequential times in the order of the arrows.*
